# Comprehensive insights from composition to functional microbe-based biodiversity of the infant human gut microbiota

**DOI:** 10.1038/s41522-023-00392-6

**Published:** 2023-05-11

**Authors:** Gabriele Andrea Lugli, Leonardo Mancabelli, Christian Milani, Federico Fontana, Chiara Tarracchini, Giulia Alessandri, Douwe van Sinderen, Francesca Turroni, Marco Ventura

**Affiliations:** 1 Laboratory of Probiogenomics, Department of Chemistry, Life Sciences, and Environmental Sustainability, University of Parma, Parma, Italy; 2 Department of Medicine and Surgery, University of Parma, Parma, Italy; 3 Microbiome Research Hub, University of Parma, Parma, Italy; 4 APC Microbiome Institute and School of Microbiology, Bioscience Institute, National University of Ireland, T12YT20 Cork, Ireland

**Keywords:** Metagenomics, Microbiome

## Abstract

During infancy, gut microbiota development is a crucial process involved in the establishment of microbe–host interactions which may persist throughout adulthood, and which are believed to influence host health. To fully understand the complexities of such interactions, it is essential to assess gut microbiota diversity of newborns and its associated microbial dynamics and relationships pertaining to health and disease. To explore microbial biodiversity during the first 3 years of human life, 10,935 shotgun metagenomic datasets were taxonomically and functionally classified. Microbial species distribution between infants revealed the presence of eight major Infant Community State Types (ICSTs), being dominated by 17 bacterial taxa, whose distribution was shown to correspond to the geographical origin and infant health status. In total, 2390 chromosomal sequences of the predominant taxa were reconstructed from metagenomic data and used in combination with 44,987 publicly available genomes to trace the distribution of microbial Population Subspecies (PS) within the different infant groups, revealing patterns of multistrain coexistence among ICSTs. Finally, implementation of a metagenomic- and metatranscriptomic-based metabolic profiling highlighted different enzymatic expression patterns of the gut microbiota that allowed us to acquire insights into mechanistic aspects of health-gut microbiota interplay in newborns. Comparison between metagenomic and metatranscriptomic data highlights how a complex environment like the human gut must be investigated by employing both sequencing methodologies and possibly supplemented with metabolomics approaches. While metagenomic analyses are very useful for microbial classification aimed at unveiling key players driving microbiota balances, using these data to explain functionalities of the microbiota is not always warranted.

## Introduction

The microbiota is represented by a complex community of microorganisms that coexist with the host and may influence host health. In mammals, the highest density of such microbial populations can be found in the intestinal tract, where they form a mix of autochthonous and allochthonous (or transient) microorganisms, which are believed to be predominantly of dietary origin^1^. During the first couple of months following birth, the infant gut microbiota is characterized by low biodiversity, being mainly populated by microorganisms belonging to the Actinomycetota and Pseudomonadota phyla (formerly named Actinobacteria and Proteobacteria)^2^. Being a member of the former phylum, *Bifidobacterium* represents the dominant bacterial genus of the healthy infant gut microbiota^3,4^. However, in the period between weaning and 3 years of life, relative abundances of members belonging to the Bacillota and Bacteroidota phyla increase (formerly named Firmicutes and Bacteroides) while that of *Bifidobacterium* diminishes, thereby shaping the infant gut microbiota into a more complex and diverse ecosystem that will accompany the host for the rest of its life^4^.

The infant gut microbial composition is influenced by many factors, such as mode of delivery, diet, and gestational age^5–7^. Comparison between natural- and cesarean section-delivered infants has revealed many compositional differences in the gut microbiota, with high prevalence of members of the genus *Lactobacillus* and *Prevotella* in vaginally born babies^8,9^. This highlights that bacteria that are naturally inherited by the mother’s microbiota by vertical transmission represent, in humans and many other mammalian species, the initiating event in early life gut microbiota formation^10^. Conversely, preterm infants or babies with severe health challenges often suffer from delayed gut colonization by commensal bacteria with a higher load of (opportunistic) pathogens, such as *Staphylococcus*, *Enterococcus*, and *Clostridium*^11^.

The human gut microbiota possesses elaborate metabolic digestion capabilities, being responsible for the degradation of complex carbohydrates, fats/lipids, and proteins, which in turn results in the production of a myriad of metabolites, which can be used by the host and which may impact on host health^12,13^.

The current study aimed to explore genome variability of bacterial taxa that constitute the infant gut microbiome employing an extensive collection of metagenomic data and related metadata gathered from multiple studies across the globe and corresponding to infants from birth until the age of 3 years. The resulting collection of 10,935 metagenomic datasets allowed the identification of key bacterial signatures of the infant microbiome that correlate with distinct community-state types. A screening of phylotypes, or, as recently defined, Population Subspecies (PS)^14^, which allows the identification of genomically identical strains, was performed among samples using thousands of metagenomically reconstructed genome sequences. Finally, metabolic reconstruction of the enrolled infant microbiomes provided insights into the functional signatures of these microorganisms that dominate the infant gut during their first years of life, and that appear to be correlated to health state, from a metagenomic and metatranscriptomic perspective.

## Results

### Detailed reconstruction of the infant gut bacterial composition

A total of 10,935 publicly available datasets, retrieved from 40 cohorts from various geographical origins, were subjected to microbial profiling based on short-read taxonomic classification down to the species level (Supplementary Table 1). Collected data were filtered based on a number of parameters as outlined in the “Methods”, to allow removal of samples that did not meet DNA quality standards required for the ensuing analyses.

**Fig. 1:**
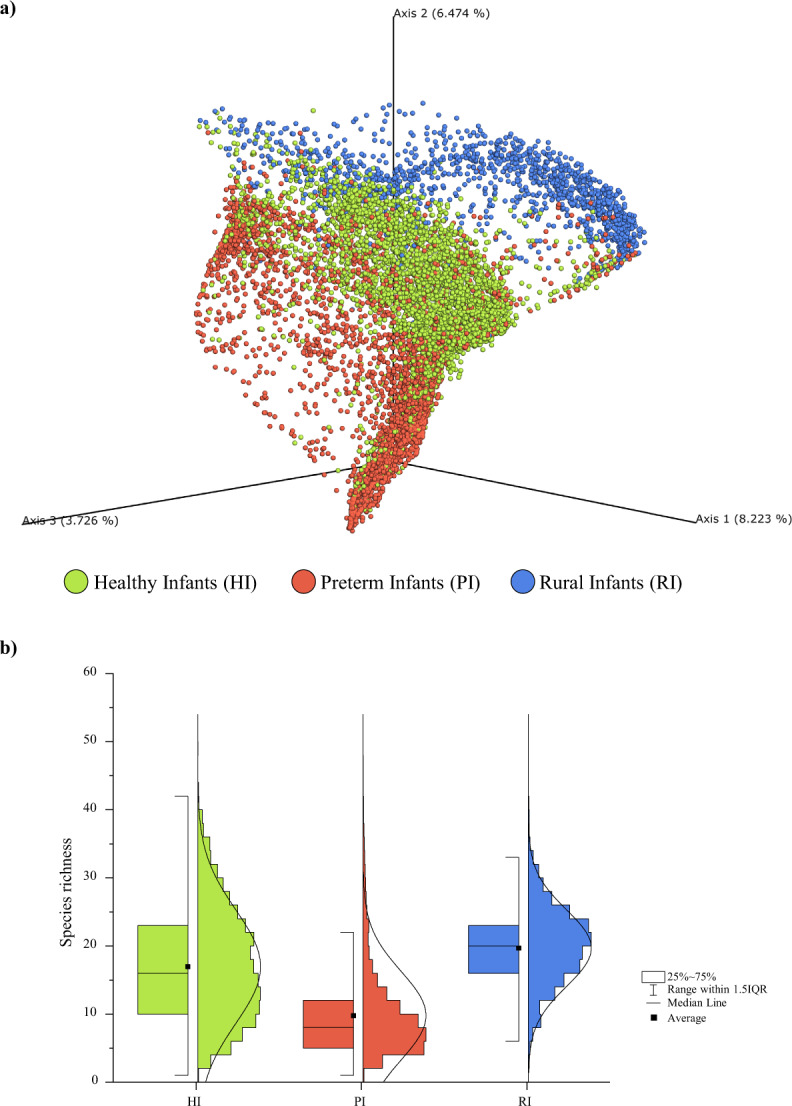
Microbial biodiversity of healthy, ill/preterm, and rural infants. **a** Displays the principal coordinate analysis (PCoA) of the collected infant samples represented in different colors by means of the three major groups. **b** Shows a box and whisker plot of the species richness calculated through the number of observed bacterial species of the three groups. The bottom and top of the box represent the first and third quartiles, and the band inside the box is the median. Moreover, the ends of the whiskers represent the 1.5 interquartile range of the sample. Source data are provided as a Source Data file.

A preliminary analysis was performed by considering all assessed samples in order to identify macrolevel correlations between samples based on their bacterial composition and metadata (Supplementary Table 1). Beta-diversity investigation represented through Principal Coordinate Analysis (PCoA) based on Bray–Curtis dissimilarity index allowed the identification of three major groups among the overall sample collection which correlated with the host health status and lifestyle, i.e., healthy infants (HI), preterm infants (PI), and rural infants (RI) (PERMANOVA *P* value of <0.05) (Fig. 1). In this context, samples belonging to HI group had been collected from healthy full-term infants belonging to urbanized countries (*n* = 4255), while PI samples had originated from preterm newborns and critically ill infants affected by necrotizing enterocolitis (NEC) or CD55 deficiency with hyperactivation of complement, angiopathic thrombosis, and severe protein-losing enteropathy (CHAPLE syndrome) also belonging to urbanized countries (*n* = 5353). Furthermore, RI samples represent a healthy infant group whose members do not inhabit an urbanized country (*n* = 1327). These findings therefore highlighted marked compositional differences of the gut microbiota between each of the three identified groups.

Species richness distribution calculated through the number of identified bacterial species with relative abundance higher than 0.05% revealed a difference in complexity between samples belonging to each of the three infant host groups (ANOVA post hoc *P* value < 0.05) (Fig. 1). These data corroborate previous studies highlighting the reduced gut microbiota biodiversity of preterm and sick infants when compared to healthy controls, as well as studies that had revealed that the gut microbial composition of newborns is different between urbanized and rural settings, in both cases conforming this at species level what had previously been observed at genus level^15–18^.

Taxonomic profiling at the species level was employed to identify microorganisms that occur at the highest prevalence among the assessed samples, revealing that *Escherichia coli* (48.5%), *Bifidobacterium longum* (36.5%), and *Enterococcus faecalis* (27.9%) are the most prevalent bacterial species, thus representing core members of the infant gut microbiota (Table 1). As expected, these species are known to be associated with early gut microbiota development and were followed in prevalence order by *Bifidobacterium breve* (24.6%) and *Bifidobacterium bifidum* (21.3%). These findings indeed confirm that the infant gut microbiota during the initial stages of life is harbored by various species that belong to the genus *Bifidobacterium*^4,19^.

**Table 1:** Prevalence of the infant gut microorganisms.

Species	Number of samples	Prevalence	Prevalence as major player^a^
***Escherichia coli***	5299	48.5%	24.0%
***Bifidobacterium longum***	3988	36.5%	19.1%
***Enterococcus faecalis***	3048	27.9%	14.5%
*Bacteroides spp*.	2808	25.7%	1.1%
***Bifidobacterium breve***	2692	24.6%	8.3%
***Bifidobacterium bifidum***	2330	21.3%	7.3%
***Klebsiella michiganensis***	2302	21.1%	5.9%
*Bifidobacterium spp*.	2282	20.9%	0.8%
***Klebsiella pneumoniae***	2088	19.1%	8.5%
***Staphylococcus epidermidis***	1934	17.7%	9.2%
*Veillonella spp*.	1873	17.1%	2.1%
*Clostridium spp*.	1836	16.8%	1.0%
***Veillonella parvula***	1802	16.5%	6.7%
***Bacteroides uniformis***	1780	16.3%	4.0%
***Ruminococcus gnavus***	1686	15.4%	4.4%
*Collinsella aerofaciens*	1660	15.2%	2.1%
***Bifidobacterium pseudocatenulatum***	1647	15.1%	3.5%
*Blautia spp*.	1587	14.5%	0.3%
***Bacteroides fragilis***	1543	14.1%	5.1%
***Blautia wexlerae***	1517	13.9%	3.3%
*Phocaeicola vulgatus*	1472	13.5%	1.9%
*Flavonifractor plautii*	1381	12.6%	1.3%
*Parabacteroides distasonis*	1374	12.6%	2.8%
*Faecalibacterium spp*.	1368	12.5%	1.2%
*Phocaeicola dorei*	1345	12.3%	2.6%
***Enterobacter hormaechei***	1342	12.3%	6.1%
***Prevotella spp****.*	1297	11.9%	8.2%
*Klebsiella spp*.	1281	11.7%	0.0%
***Prevotella copri***	1278	11.7%	8.0%
*Klebsiella variicola*	1228	11.2%	2.6%
*Veillonella atypica*	1207	11.0%	2.8%
*Klebsiella quasipneumoniae*	1199	11.0%	2.4%
*Streptococcus salivarius*	1196	10.9%	1.9%
*Ruminococcus spp*.	1168	10.7%	0.1%
*Streptococcus spp*.	1168	10.7%	1.2%
*Bifidobacterium catenulatum*	1143	10.5%	1.3%
*Faecalibacterium prausnitzii*	1103	10.1%	0.7%

^a^Prevalence within samples when identified among the three most abundant species.

Bold species possessed >10% Prevalence in conjunction than >3% Prevalence as major player.

### Delineating the core microbial species residing in the infant’s gut

**Fig. 2:**
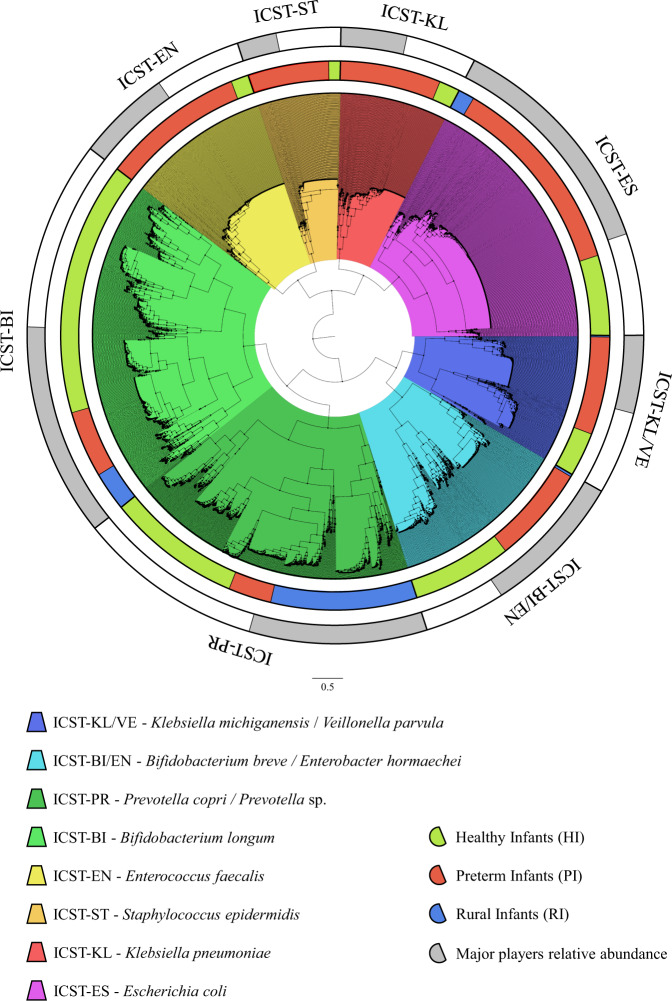
Representation of the infant community-state types (ICSTs). The circular cladogram illustrates the eight ICSTs, highlighted in different colors, obtained by means of hierarchical clustering (HCL) analysis based on the bacterial relative abundances between samples. Source data are provided as a Source Data file.

**Fig. 3:**
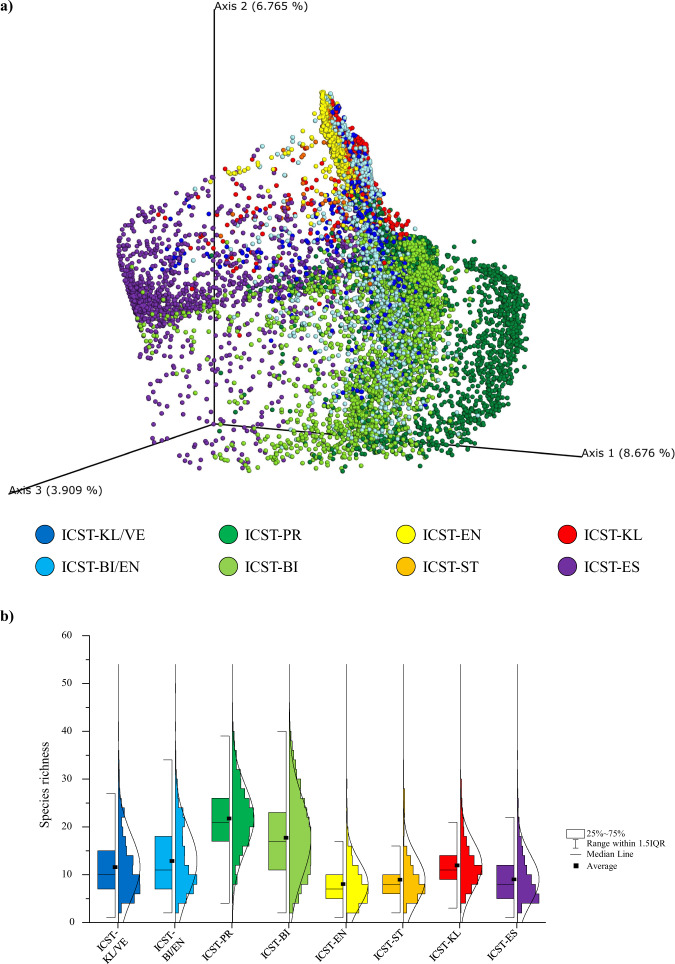
Microbial biodiversity of ICSTs. **a** Shows the principal coordinate analysis (PCoA) of the collected infant samples represented in different colors by means of the eight ICSTs. **b** Shows a box and whisker plot of the species richness calculated through the number of observed bacterial species of the eight groups. The bottom and top of the box represent the first and third quartiles, and the band inside the box is the median. Moreover, the ends of the whiskers represent the 1.5 interquartile range of the sample. Source data are provided as a Source Data file.

Recent studies of the infant gut microbiota have attempted to identify specific infant enterotypes, also known as gut community-state types (CSTs), by detecting distinct clusters of recurring microbial taxa based on genus-level classification of the gut microbiota^5,20^. The availability of a complete shotgun metagenomic database encompassing 10,935 infant gut microbiomes allowed us to provide a detailed classification of microorganisms at the species level (Supplementary Fig. 1). An investigation of infant community-state types (ICSTs) was performed by cluster analysis through hierarchical clustering (HCL) of the microbial composition of the included samples (Fig. 2). In this context, only clusters supported by at least 500 infants were investigated in detail to maximize the robustness of these analyses. Moreover, to identify ICSTs, only species that were identified at a prevalence higher than 10% among infants were considered (see “Methods”) (Table 1), and clusters were named according to the species that was shown to be present at the highest relative abundance (Fig. 2). The resulting ICSTs were further validated by PCoA analysis and PERMANOVA (*P* value < 0.05) (Fig. 3).

In silico analyses of the assessed samples allowed the identification of eight ICSTs (Fig. 2), constituted by at least 500 infants, which were named based on the dominant species as reported in Table 2, i.e., ICST-KL/VE (*Klebsiella michiganensis/Veillonella parvula*), ICST-BI/EN (*Bifidobacterium breve/Enterococcus hormaechei*), ICST-PR (*Prevotella copri*), ICST-BI (*Bifidobacterium longum*), ICST-EN (*Enterococcus faecalis*), ICST-ST (*Staphylococcus epidermidis*), ICST-KL (*Klebsiella pneumoniae*), and ICST-ES (*Escherichia coli*). Notably, the ICST-PR (*n* = 1981) and ICST-BI (*n* = 2198) were mainly represented by HI and RI (88% and 81%, respectively), while ICST-BI/EN (*n* = 1216) represented samples from HI and PI in essentially equal distribution (50% and 49%, respectively) (Fig. 2 and Supplementary Fig. 1). Interestingly, ICST-PR was populated by multiple *Prevotella* species which, alongside *Prevotella copri*, were identifiable as yet unclassified species. The remaining five ICSTs were found to be predominantly represented by samples from PI, i.e., ICST-ES (*n* = 1818), -EN (*n* = 980), -KL (*n* = 735), -ST (*n* = 569), and -KL/VE (*n* = 873) (Fig. 2 and Supplementary Fig. 1). Interestingly, all RI were distributed among ICST-PR, -BI, and -ES, representing the most populated ICSTs in terms of the included number of infant microbiome datasets (Fig. 2). This finding suggests that the latter ICSTs represent pre-industrial infant gut microbiomes^21^. Based on this notion, other identified ICSTs may have been established more recently in infants of urbanized countries representing industrialized gut microbiomes. Furthermore, microbiome compositional analysis revealed an association with an apparent insurgence of opportunistic pathogens in “modern” ICSTs, since they were mainly constituted by *Enterococcus faecalis*, *Klebsiella michiganensis*, *Klebsiella pneumoniae*, and *Staphylococcus epidermidis*.

**Table 2:** Infant Community State Types (ICSTs) composition.

ICST Code	Representative species	Number of infants	HI	PI	RI	Correlation with host health
ICST-BI	*Bifidobacterium longum*	2198	68%	19%	14%	Healthy-ICST
ICST-PR	*Prevotella copri Prevotella* spp.	1981	42%	12%	46%	Healthy-ICST
ICST-ES	*Escherichia coli*	1818	29%	65%	6%	Unhealthy-ICST
ICST-BI/EN	*Bifidobacterium breve*	1216	50%	49%	1%	Mixed-ICST
	*Enterobacter hormaechei*					
ICST-EN	*Enterococcus faecalis*	980	15%	85%	0%	Unhealthy-ICST
ICST-KL/VE	*Klebsiella michiganensis*	873	31%	69%	1%	Unhealthy-ICST
	*Veillonella parvula*					
ICST-KL	*Klebsiella pneumoniae*	735	17%	83%	0%	Unhealthy-ICST
ICST-ST	*Staphylococcus epidermidis*	569	13%	87%	0%	Unhealthy-ICST

The above-reported distributions revealed that ICST-PR and -BI were dominated by disease-free infants representing healthy-correlated ICSTs (Healthy-ICST) with a predominance of *Bifidobacterium longum* and *Prevotella copri* which have previously been considered as commensal colonizers of the infant gut^18,19^. Accordingly, ~90% of RI were distributed among the latter ICSTs, thus being an integral part of Healthy-ICST (Fig. 2). On the other hand, ICST-KL/VE, -EN, -ST, -KL, and -ES mainly represented PI samples, ranging from 65% in ICST-ES to 87% in ICST-ST, corresponding to preterm- and disease-associated ICSTs (Unhealthy-ICST) that encompass high levels of opportunistic pathogens. Furthermore, ICST-BI/EN does not appear to associate with either of these two major host subdivisions as they represent a mixed-ICST (Mixed-ICST) with more or less equal numbers of healthy or preterm/sick babies.

Reported ICSTs were further validated by their species richness distribution, supporting a difference in complexity between ICSTs (ANOVA post hoc *P* value < 0.05) (Fig. 3). In detail, high microbial biodiversity was reported for ICST-PR, -BI, and -BI/EN, corroborating the notion that a highly diverse infant gut microbiota is correlated with the presence of species belonging to the genera *Prevotella* or *Bifidobacterium*^18,19^. Conversely, ICST-EN, -ST, -ES, and -KL, i.e., the Unhealthy-ICST, corresponding to premature or ill infants, were characterized by lower microbial biodiversity, when compared to the Healthy-ICST^16^, suggesting an association with antibiotic usage (based on metadata antibiotics had been administered to 9% of infants belonging to the PI group; Supplementary Table 1).

### Distribution of the core microbial species based on infant mode of delivery, feeding, antibiotic exposure, and age

Collected metadata allowed us to define a correlation between core microbial species distribution in the infant gut and specific factors, such as mode of delivery, feeding, antibiotic exposure, and age. For example, comparing gut microbiomes of cesarean (*n* = 672) and vaginally (*n* = 1012) delivered infants highlighted statistically significant correlations of *Bacteroides uniformis*, *Bacteroides fragilis*, and *Escherichia coli* to the vaginal delivery (Supplementary Table 2). On the other hand, *Ruminococcus gnavus*, *Veillonella parvula*, *Enterobacter hormaechei*, *Klebsiella pneumoniae*, and *Enterococcus faecalis* were found to be significantly more abundant in the cesarean-delivered infants. Interestingly, none of the *Bifidobacterium* and *Prevotella* species were observed to correlate with the two delivery modes, resulting only in a slight increase of *Bifidobacterium bifidum* and *Bifidobacterium breve* average abundance in vaginally delivered infants.

From a feeding perspective, formula-fed infants (supplied with a breast milk substitute) (*n* = 276) positively correlate with *Enterobacter hormaechei* and *Enterococcus faecalis*, while several microbial species were found to be significantly higher in abundance among breast-fed (*n* = 372) and mixed-fed (supplied with both breast milk and substitute) (*n* = 661) infants (Supplementary Table 2). Specifically, the latter infants were characterized by an increase of all four core bifidobacterial species represented by *Bifidobacterium longum*, *Bifidobacterium breve*, *Bifidobacterium bifidum*, and *Bifidobacterium pseudocatenulatum*.

A similar profile was also observed analyzing infants receiving an antibiotic therapy (*n* = 1082), and infants reported not being treated with antibiotics (*n* = 948). In the former group, a significant increase in relative abundance of *Blautia wexlerae*, *Staphylococcus epidermidis*, and *Enterococcus faecalis* was observed (Supplementary Table 2). In contrast, the microbiome of antibiotic-free infants was associated with several health-related microorganisms, such as *Prevotella copri* and the four breast-fed-associated bifidobacterial species.

Finally, an investigation focusing on infant aging allowed profiling microbiomes at infants at different ages, i.e., one (3–365 days, *n* = 8343), two (366–730 days, *n* = 525), and 3 years (732–1162 days, *n* = 143). This analysis revealed an expanded microbial diversity of the gut microbiota of infants over the age of one with an increased abundance of *Blautia wexlerae*, *Bacteroides uniformis*, *Bacteroides fragilis*, and *Prevotella copri* (Supplementary Table 2). Conversely, except for *Ruminococcus gnavus*, all other core microbial species were significantly abundant in the first year of life.

Altogether, our metadata analyses highlighted that the core *Bifidobacterium* species were positively associated with the first year of childhood in antibiotic-free and breast- or mixed-fed infants (Supplementary Table 2). Notably, the mode of delivery does not significantly affect the average abundance of bifidobacteria or *Prevotella copri*. Instead, the latter species was found to be most abundant in 3-year-old infants, highlighting an opposite trend with respect to bifidobacteria that were found to reduce in relative abundance with increasing age. Furthermore, opportunistic bacteria such as *Klebsiella michiganensis* and *Klebsiella pneumoniae* tend to disappear beyond the age of one. The same trend was observed for *Staphylococcus epidermidis*, which was completely absent from the microbiome of infants older than 1 year, highlighting how the probable contamination of this species from the mother’s skin does not longitudinally fit in the gut environment.

### Strain-specific variability of the infant gut microbiota

To trace the intraspecies variability of the 17 most abundant microbial taxa identified amongst ICSTs, species-specific databases were constructed encompassing 44,987 publicly available genomes downloaded from the NCBI database (Supplementary Table 3). In addition, to enrich the latter databases, metagenomically reconstructed genome sequences were included from 1,700 samples with the highest abundance of microbial core elements. In this manner a total of 2390 genomes were reconstructed with a completeness level higher than 50 %, together with a contamination level below 1.95% (see “Methods”). One or more unclassified species of the *Prevotella* genus appeared to be present as a major constituent of the ICST-PR, and indeed whole-metagenome assemblies allowed the recovery of metagenomic contigs corresponding to a single putative novel species. Among the 98 assembled genomes belonging to this putative novel taxon, 86 *Prevotella* sp. showed an average nucleotide identity (ANI) above 95%, thus highlighting an unclassified *Prevotella* species which appears to be highly prevalent and abundant in the ICST-PR (Supplementary Table 4). Interestingly, 97% of *Prevotella* spp. genome sequences were reconstructed from RI metagenomes, highlighting a correlation with the geographical metadata. Nonetheless, the genome reconstruction and validation of this putative novel species was also detected in three different studies involving urbanized infants, thus confirming its presence in Western world populations. Subsequently, validated *Prevotella* sp. genomes were also included in the phylotype/PS profiling together with bacterial genomes of 16 other predominant taxa (Table 1). The collected 47,377 chromosomal sequences were employed to build 17 non-redundant databases of species-specific *k*-mers by clustering genomes with >99.8% of sequence identity, allowing the identification of each species across the 10,935 infant gut microbiomes and revealing their distribution at the PS level among samples^14^.

As expected, the retrieved prevalence at species level confirmed data obtained in the taxonomic assignment of the reads (Table 3 and Supplementary Fig. 1). Focusing on the phylotype/PS level, among Healthy-ICST, the highest strain richness within a species was predicted to belong to *Prevotella copri* and *Prevotella* spp. (average of 2.5 and 2.9 PS, respectively), followed by *Bifidobacterium longum* (average of 1.9 PS when present) (Table 3). In contrast, among species belonging to the Unhealthy-ICST, the highest strain richness was predicted to belong to *Staphylococcus epidermidis* (1.9 PS when present), highlighting the impact of cesarean section delivery routinely performed to mothers of preterm infants populating Unhealthy-ICST and the correlated contamination by skin-harbored bacteria on the gut microbiota of infants, resulting in multiple *Staphylococcus epidermidis* PS contaminants in 43.7% of the samples (Table 3). On the other hand, the highest strain richness detected when multiple PS coexist in the same environment belonged to *Bifidobacterium longum*, retrieved in 34.7% of Healthy-ICST individuals harboring two PS, and in 13.2% of Healthy-ICST members sharing three PS (Table 3). In contrast, the highest value of strain richness detected when at least two PS coexist in Unhealthy-ICST was represented by *Enterococcus faecalis* in 12.4% of the samples. Thus, the wide-ranging coexistence of multiple *Bifidobacterium longum* PS in Healthy-ICST reflects a multistrain coexistence associated with HI, being absent or undetectable in the microbiome of preterm/unhealthy infants. The PS distribution between HI, PI, and RI was also explored to validate the latter assumptions, showing several different phylotype distributions (Supplementary Table 5). In this regard, RI harbors the highest species richness of *Prevotella copri* and *Prevotella* spp. (average of 2.7 and 3 PS, respectively), and the highest strain richness was detected when multiple PS coexist in the same environment (*Bifidobacterium longum*, retrieved in 38.8% of RI individuals harboring two PS, and in 19.1% of RI members sharing three PS). Thus, a large part of the Healthy-ICST PS biodiversity observed for *Bifidobacterium* and *Prevotella* species was corresponding to non-urbanized infants, exhibiting the highest multistrain coexistence of multiple commensal bacteria (Supplementary Table 5).

**Table 3:** Population subspecies profiles.

Species	N° strains matched	Strains with single hit	Strain richness					Richness (if present)	Richness (all)	Prevalence
			1×	2×	3×	4×	5×			
**Healthy-ICST**										
*Bacteroides fragilis*	157	13	24.7%	6.6%	0.3%	0.0%	0.0%	1.2	0.39	31.7%
*Bacteroides uniformis*	174	32	19.1%	4.6%	0.3%	0.0%	0.0%	1.2	0.29	24.0%
*Bifidobacterium bifidum*	163	12	29.8%	13.2%	0.4%	0.0%	0.0%	1.3	0.58	43.4%
*Bifidobacterium breve*	150	34	24.5%	7.6%	0.4%	0.0%	0.0%	1.3	0.41	32.5%
*Bifidobacterium longum*	388	90	22.1%	34.7%	13.2%	1.5%	0.1%	1.9	1.37	71.5%
*Bifidobacterium pseudocatenulatum*	137	27	27.4%	4.4%	0.2%	0.0%	0.0%	1.2	0.37	32.0%
*Blautia wexlerae*	160	24	21.5%	6.9%	0.9%	0.1%	0.0%	1.3	0.38	29.4%
*Enterobacter hormaechei*	72	30	5.5%	1.0%	0.2%	0.1%	0.0%	1.2	0.08	6.8%
*Enterococcus faecalis*	114	43	11.8%	3.4%	1.1%	0.3%	0.0%	1.4	0.23	16.6%
*Escherichia coli*	1059	482	35.2%	19.5%	6.7%	1.3%	0.2%	1.6	1.00	62.8%
*Klebsiella michiganensis*	76	38	5.2%	0.6%	0.0%	0.0%	0.0%	1.1	0.06	5.7%
*Klebsiella pneumoniae*	233	107	10.3%	3.6%	0.6%	0.1%	0.0%	1.4	0.20	14.5%
*Prevotella copri*	96	8	7.5%	7.0%	6.3%	4.0%	2.4%	2.5	0.69	27.2%
*Prevotella unknown species*	59	1	3.0%	4.4%	5.9%	3.7%	2.4%	2.9	0.57	19.5%
*Ruminococcus gnavus*	176	31	22.7%	11.3%	2.0%	0.3%	0.0%	1.4	0.52	36.2%
*Staphylococcus epidermidis*	87	36	3.6%	2.1%	0.7%	0.4%	0.1%	1.7	0.12	6.8%
*Veillonella parvula*	86	12	12.9%	4.4%	0.4%	0.0%	0.0%	1.3	0.23	17.7%
**Mixed-ICST**										
*Bacteroides fragilis*	67	37	7.3%	2.2%	0.1%	0.0%	0.0%	1.2	0.12	9.6%
*Bacteroides uniformis*	57	36	8.5%	0.4%	0.0%	0.0%	0.0%	1.0	0.09	8.9%
*Bifidobacterium bifidum*	123	42	16.5%	11.4%	0.1%	0.0%	0.0%	1.4	0.40	28.1%
*Bifidobacterium breve*	134	27	26.3%	19.8%	4.9%	0.2%	0.0%	1.6	0.81	51.2%
*Bifidobacterium longum*	204	98	14.7%	15.6%	3.1%	0.2%	0.0%	1.7	0.56	33.7%
*Bifidobacterium pseudocatenulatum*	57	22	8.1%	2.4%	0.7%	0.2%	0.0%	1.4	0.16	11.4%
*Blautia wexlerae*	59	31	8.6%	1.6%	0.2%	0.0%	0.0%	1.2	0.12	10.3%
*Enterobacter hormaechei*	94	23	32.8%	10.0%	0.9%	0.7%	0.0%	1.3	0.58	44.3%
*Enterococcus faecalis*	95	36	33.5%	11.7%	2.1%	0.2%	0.1%	1.4	0.64	47.5%
*Escherichia coli*	291	175	25.5%	14.7%	3.0%	0.6%	0.2%	1.5	0.67	44.0%
*Klebsiella michiganensis*	36	14	8.5%	1.6%	0.0%	0.0%	0.0%	1.2	0.12	10.0%
*Klebsiella pneumoniae*	119	55	18.0%	5.9%	0.5%	0.0%	0.1%	1.3	0.32	24.5%
*Prevotella copri*	18	13	1.3%	0.5%	0.1%	0.1%	0.0%	1.5	0.03	2.0%
*Prevotella unknown species*	13	13	0.2%	0.3%	0.1%	0.0%	0.0%	1.9	0.01	0.6%
*Ruminococcus gnavus*	89	33	9.9%	7.2%	1.2%	0.0%	0.0%	1.5	0.28	18.4%
*Staphylococcus epidermidis*	73	23	17.5%	6.6%	3.0%	3.0%	0.8%	1.8	0.56	30.9%
*Veillonella parvula*	77	14	19.8%	5.0%	0.7%	0.0%	0.0%	1.3	0.32	25.6%
**Unhealthy-ICST**										
*Bacteroides fragilis*	65	38	2.3%	0.3%	0.0%	0.0%	0.0%	1.1	0.03	2.6%
*Bacteroides uniformis*	72	42	3.2%	0.2%	0.0%	0.0%	0.0%	1.1	0.04	3.4%
*Bifidobacterium bifidum*	76	29	2.6%	0.9%	0.0%	0.0%	0.0%	1.3	0.04	3.5%
*Bifidobacterium breve*	91	33	4.2%	1.8%	0.1%	0.0%	0.0%	1.3	0.08	6.1%
*Bifidobacterium longum*	198	77	4.2%	4.4%	1.0%	0.1%	0.0%	1.7	0.17	9.8%
*Bifidobacterium pseudocatenulatum*	37	19	1.8%	0.3%	0.0%	0.0%	0.0%	1.1	0.02	2.1%
*Blautia wexlerae*	44	19	1.9%	0.3%	0.0%	0.0%	0.0%	1.1	0.02	2.1%
*Enterobacter hormaechei*	92	27	10.0%	1.3%	0.0%	0.0%	0.0%	1.1	0.13	11.4%
*Enterococcus faecalis*	158	45	33.6%	12.4%	3.0%	0.4%	0.0%	1.4	0.69	49.4%
*Escherichia coli*	576	310	30.4%	9.3%	2.2%	0.5%	0.1%	1.4	0.59	42.7%
*Klebsiella michiganensis*	92	18	15.2%	2.4%	0.1%	0.0%	0.0%	1.2	0.20	17.7%
*Klebsiella pneumoniae*	257	78	19.7%	8.1%	1.9%	0.6%	0.0%	1.5	0.44	30.2%
*Prevotella copri*	21	14	0.6%	0.0%	0.0%	0.0%	0.0%	1.1	0.01	0.6%
*Prevotella unknown species*	15	9	0.2%	0.1%	0.0%	0.0%	0.0%	1.4	0.00	0.3%
*Ruminococcus gnavus*	78	25	2.5%	1.5%	0.1%	0.0%	0.0%	1.4	0.06	4.2%
*Staphylococcus epidermidis*	133	27	21.5%	11.8%	6.1%	2.4%	2.0%	1.9	0.83	43.7%
*Veillonella parvula*	93	11	12.8%	5.2%	1.3%	0.1%	0.0%	1.4	0.28	19.5%

**Fig. 4:**
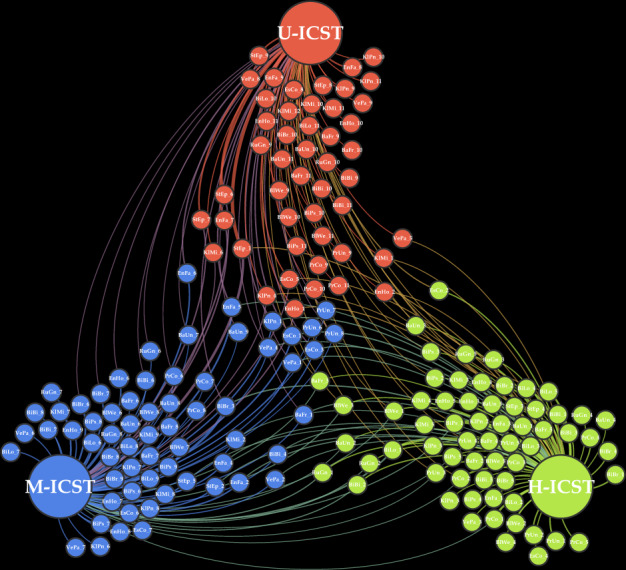
Network analysis based on the co-occurrence and co-exclusion of Population Subspecies (PS). The three large circles represent Healthy-ICST (H-ICST), Unhealthy-ICST (U-ICST), and Mixed-ICST (M-ICST) groups, while each dot denotes different PS belonging to one of the 17 dominant taxa. The bacterial scientific names are indicated as abbreviations using the first two characters of the genus and species names, e.g., BaFr *Bacteroides fragilis*, BiBi *Bifidobacterium bifidum,* EsCo *Escherichia coli*, PrCo *Prevotella copri*. Source data are provided as a Source Data file.

To identify associations between prevalent PS and ICSTs, a network based on their relationships was produced (Fig. 4), highlighting that 66% of the highly prevalent PS (five most prevalent strains per species) were exclusive to either Healthy-ICST, Unhealthy-ICST, or Mixed-ICST. *Bacteroides fragilis*, *Blautia wexlerae*, *Klebsiella michiganensis*, and *Klebsiella pneumoniae* PSs showed the highest ICST specificity, with more than 80% being highly prevalent in a single ICST. In contrast, *Enterococcus faecalis*, *Escherichia coli*, and *Staphylococcus epidermidis* PS showed the lowest ICST specificity (Fig. 4). Interestingly, 20 out of 175 analyzed PS were identified in members of each infant group, of which *Escherichia coli* PS (*n* = 3) was shown to be the most prevalent. In contrast, *Bacteroides uniformis*, *Bifidobacterium bifidum*, *Klebsiella michiganensis*, and *Ruminococcus gnavus* PS were shared between the three main ICSTs, with no ICST-specific correlations (Fig. 4). Notably, when investigating shared PS between HI, PI, and RI, the number of PS dropped from 20 to 4, highlighting the existence of a peculiar PS distribution within the gut microbiome of RI that was absent in the urbanized population.

Altogether, the PS analysis revealed that Healthy-ICST, Unhealthy-ICST, and Mixed-ICST showed a unique profile of highly prevalent PS, indicating that a correlation exists between infant health status and specific PS, and revealing patterns of multistrain coexistence among particular ICSTs. Furthermore, the uniqueness of the identified phylotypes was even more emphasized between HI, PI, and RI, revealing a correlation between urbanization and PS. Identified PS specificity uncovered how certain phylotypes are directly correlated with host health and point to their potential use as biomarkers.

### Metagenomic-based overview of the infant microbiota metabolic capabilities

**Fig. 5:**
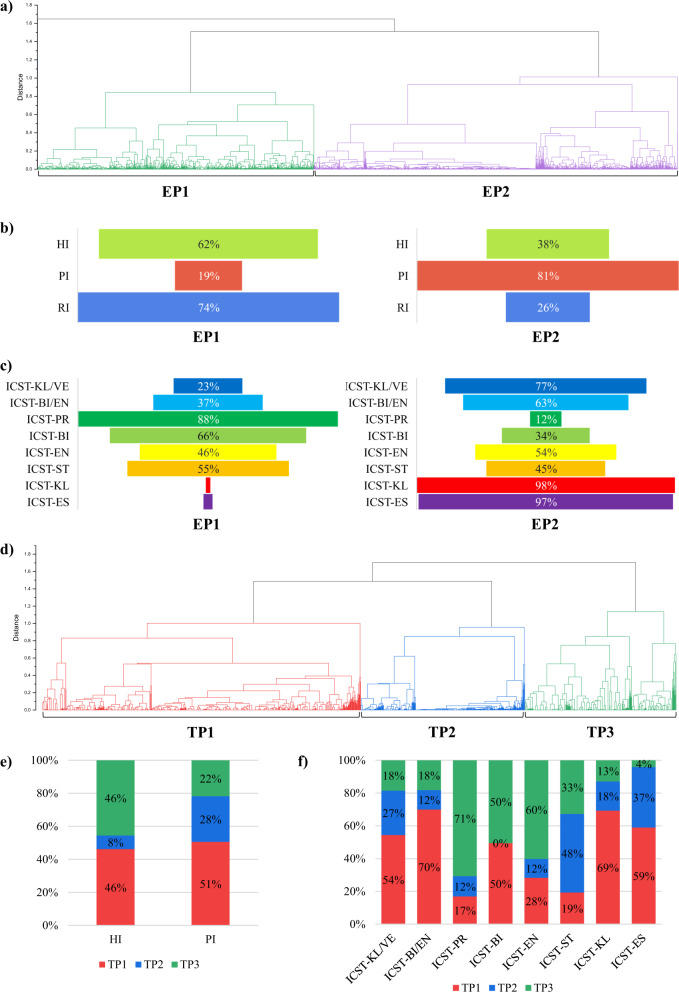
Enzymatic profiling of metagenomic and metatranscriptomics datasets. **a** Exhibits a clustering based on the metagenomic enzymatic profiling of each microbiome validated through a silhouette analysis. **b** Displays the distribution of HI, PI, and RI in the two clusters EP1 and EP2, while panel **c** reports the distribution of the eight ICSTs between clusters. **d** Shows the respective subdivision in clusters based on the metatranscriptomics enzymatic profiling. **e** Describes distribution of HI, PI, and RI in the three clusters TP1, TP2, and TP3, while panel **f** displays the distribution of the eight ICSTs between clusters. Source data are provided as a Source Data file.

The infant gut metagenomic datasets used for microbial taxonomic profiling were also explored to define their metabolic capabilities. Thus, a detailed investigation based on the predicted enzymatic activities of the infant gut microbiota was performed to gain a precise view of the metabolic potential encoded by the predicted ICSTs. Overall, 2062 different enzymes, classified based on Enzyme Commission (EC) categories^22^, were identified across the datasets using the Metacyc database as ref. ^23^. Data consistency was then validated with a silhouette clustering method, facilitating a matching exercise of microbiomes with a similar enzymatic profile (EP) and unveiling two robust clusters defined as EP1 and EP2 (Fig. 5).

Looking at the metadata, 62% of HI and 74% of RI samples were assigned to EP1, while 81% of the PI-associated datasets were shown to correspond to EP2, highlighting a distribution that largely correlates with infant health status (Fig. 5). Even more significant was the distribution of the eight ICSTs (as based on the microbial community’s taxonomy) among two functional clusters EP1 and EP2. Specifically, 98% and 97% of members of the ICST-KL and ICST-ES, respectively, were allocated in EP2, followed by ICST-KL/VE (77%) and ICST-BI/EN (63%) (Fig. 5). In contrast, 88% and 63% of members of the ICST-PR and ICST-BI, respectively, were shown to correspond to EP1, disclosing a marked association between Healthy-ICSTs with EP1 (78%) and Unhealthy-ICSTs in EP2 (80%) (Fig. 5).

A multivariable association analysis conducted by Maaslin2, including multiple covariates (see “Methods”), was then applied to investigate association between enzymatic reactions and EPs, revealing a significant association of 586 enzymatic reactions to EP1 and 876 to EP2 (Supplementary Table 6). The larger number of enzymatic reactions in EP2 is explained by a higher number of different enzyme families classified as oxidoreductases (69%), transferases (57%), hydrolases (58%), lyases (66%), isomerases (59%), and translocases (80%) when compared to EP1 (Fig. 5). Generally, the microbiota of infants that fall into the EP2 seem to possess a more extensive number of metabolic capabilities correlating with the Unhealthy-ICST, which includes ICST-KL and ICST-ES. This finding may be due to the much larger pangenome of *Escherichia* (mean number of genes = 4648) and *Klebsiella* (mean number of genes = 5153) when compared to the other main ICSTs taxa, such as *Bifidobacterium* (mean gene no. = 1860) and *Prevotella* (mean gene no. = 2460).

Statistically significant correlations between ICSTs and 495 enzymatic reactions associated to 142 key compounds known to be associated with host health^12,13^ uncovered several routes in the production and degradation of metabolites (Supplementary Table 7). Taurine and cadaverine production as well as the metabolism of tryptophan and putrescine derivates were positively associated with EP2 and Unhealthy-ICST, in particular ICST-KL and ICST-ES (Supplementary Table 7), highlighting disease-associated microorganisms correlated with intestinal inflammation^24–26^. Additional compounds associated with microbiome dysbiosis, such as polyamines like 3-aminopropanal and N1-acetylspermidine, production of L-carnitine, methanol, and succinate, were positively correlated to EP2^27,28^. As reported in Supplementary Table 7, many other metabolites that are positively linked with host health were shown to be unidirectionally correlated with EP2 instead of EP1, being consistent with the high number of distinct enzyme families associated with EP2.

Altogether, the metabolic pathway prediction of EP2 members highlighted the expanded metabolic abilities of the Unhealthy-ICST when compared to the Healthy-ICST, likely reflecting the presence of opportunistic bacteria encoding an extensive enzymatic repertoire for metabolite production, including compounds correlated with intestinal inflammation.

### Metabolic abilities of the infant microbiota from a metatranscriptomics perspective

Datasets of 1602 infant gut metatranscriptomes were assessed to explore expression of predicted enzymatic activities (Supplementary Table 8). The infant gut transcriptomic data was processed as previously reported for metagenomic data, with an additional filtering step to remove sequences belonging to microbial rRNA and tRNA genes. The silhouette clustering method matched microbiomes with a similar transcriptomic enzymatic profile (TP), unveiling three robust clusters defined as TP1, TP2, and TP3, not corresponding to the previous subdivision in two EP as assessed by the metagenomic enzymatic screening (Fig. 5).

Although the number of analyzed transcriptome samples is substantially less than the metagenomic dataset used for the enzymatic screening, the eight ICSTs were all well represented by at least 65 samples allowing a transcriptome-based investigation of ICST-specific or taxon-specific transcription profiles. Species distribution was evaluated through microbial profiling based on short-read taxonomic classification, and the ICST distribution was based on the average abundance of the core microbiota. Interestingly, no specific predominance of samples related to an ICST was reported among TPs since none of the analyzed clusters was overrepresented by ICSTs that form the Healthy-ICST or Unhealthy-ICST (Fig. 5). This finding was in contrast with above-reported sample distribution for the metagenomic enzymatic profiling, which correlates with the distribution of the main bacterial constituents.

A multivariate association analysis between TPs and the associated (predicted) enzymatic functions highlighted that TP1 and TP2 represented 1262 and 1020 significant positive associations with EC numbers, respectively (Supplementary Table 9). In contrast, TP3 revealed just 648 significant positive associations, which may reflect the higher number of Healthy-ICSTs associated with this TP when compared to TP1 and TP2. Likewise, the metagenome enzymatic screening of EP1 revealed that the Healthy-ICST was associated with a lower number of distinct enzymatic reactions (Supplementary Table 6). Nonetheless, profiling of significant correlations between ICSTs and the transcriptome-based enzymatic reactions revealed an unexpected pattern in the metatranscriptomics analysis, which did not appear to be in agreement with metagenomics data.

The covariance assessment between ICSTs and 142 key compounds impacting on human health revealed that the majority of the positive correlations of ICSTs encompassing the Unhealthy-ICST were not verified in metatranscriptomics data, highlighting a differential expression with respect to the predicted enzymatic capability of microbial taxa inhabiting the infant’s gut (Supplementary Table 10). Furthermore, ICST members of the Healthy-ICST were shown to exhibit a positive correlation with respect to biosynthesis of amino acids, such as arginine, glycine, aspartate, leucine, asparagine, and phenylalanine, and biosynthesis of alpha-ketoisovaleric acid, a precursor of valine. In addition, quinolinic acid, deoxyxylulose-5-phosphate, two precursors for vitamins, were also unidirectionally correlated with the Healthy-ICST. These latter enzymatic reactions were not positively correlated with Healthy-ICST in our enzymatic analysis based on metagenomic data probably due to high gene redundancy of pathogenic bacteria constituting the Unhealthy-ICST.

Despite the extensive metabolic ability as profiled by the enzymatic metagenomic analyses of opportunistic bacteria encompassing the Unhealthy-ICST (Supplementary Table 7), their metatranscriptomes revealed a completely different enzymatic profile (Supplementary Table 10). These results indicate that the metagenomic data represent biochemical and functional potential of the microbial species inhabiting the infant’s gut, while metatranscriptomic data allowed us to reveal which genes play an active role among these microbial communities. For example, significant compounds associated with individual ICSTs, such as deoxyxylulose-5-phosphate (ICST-PR), l-asparagine (ICST-BI), glycerol (ICST-EN and ICST-ST), S-adenosylhomocysteine (ICST-KL), 4-hydroxyphenylpyruvic acid, and glutathione (ICST-ES) were identified (Supplementary Table 10).

### Limitations and potential applications of this study

One limitation of the present survey relates to the dataset distribution. Since our metagenomic and metatranscriptomic analyses rely on DNA sequenced from other studies, we can only assume that the observed diversity in terms of microbial distribution and enzymatic reactions represents a comprehensive and true reflection of reality. This limitation is even more substantial for the metatranscriptomic screening, which exhibits less variability in terms of metadata included in this study. Future infant gut microbiome investigations from additional countries may expand the overall scientific scenario proposed by this study. Furthermore, in this work, we focus our metagenomic and metatranscriptomic analyses on 142 compounds that have recently been correlated with human health. Many other compounds have not been taken into consideration with as many enzymatic reactions that may be useful to expand our knowledge about host-microbe interactions. For example, these data can be used to evaluate the carbohydrate metabolism of the microbiome and its associated contribution to infant health, however, validation of the inferred enzymatic profiles should be achieved through additional in vitro experiments.

A potential application of future metagenomic and metatranscriptomic analyses of the infant gut microbiota will be to correlate the profiled metabolic pathways to specific clinical outcomes. A detailed metabolic map derived from expressed genes may guide appropriate treatment of infant patients through a personalized medicine approach that is not only based on host genetics but also on the patient’s corresponding microbiota.

## Discussion

In this work, we provided a comprehensive map of the gut microbiota composition of infants encompassing more than ten thousand datasets corresponding to healthy, preterm, and rural infants. The analysis performed at species level revealed 17 bacterial players that are highly prevalent among the analyzed samples and that make up eight statistically supported clusters named infant community-state types (ICSTs). *Bifidobacterium longum* and *Prevotella copri* were dominant in disease-free infants representing ICSTs that were assigned a healthy status (Healthy-ICST) together with a novel, yet unclassified species belonging to the *Prevotella* genus. The high prevalence of this *Prevotella* spp. in Healthy-ICST may reflect an unknown microorganism with an as yet unknown, yet important role in infant health. In addition, *Bifidobacterium longum*, *Bifidobacterium breve*, *Bifidobacterium bifidum*, and *Bifidobacterium pseudocatenulatum* were positively associated with antibiotic-free and breast-fed infants who are less than a year old of age, while the mode of delivery does not affect the relative abundance of *Bifidobacterium* and *Prevotella* species. Interestingly, while the relative abundance of bifidobacterial species tends to decrease in infants older than 1 year, *Prevotella* showed an opposite trend with its higher relative abundance in 3-year-old infants, highlighting a shift to a more mature microbiota. Notably, the *Prevotella* ICST distribution among infants shifts from 18% at the age of 1 year to 40% and 61% at 2 and 3 years of age, respectively, unveiling the significance of *Prevotella* not only in RI, but also in infants of urbanized countries. Furthermore, the existence of the putative novel *Prevotella* species described above was validated through its genome reconstruction from 86 infants primarily correlating its occurrence in RI.

The genome reconstruction of several thousand microbial genomes allowed an exploration at PS level, i.e., identifying genomically distinct strains of a given species among samples, and their distribution among ICSTs. Despite the fact that specific taxa dominated each ICST, many species were distributed among ICSTs at a lower abundance, allowing us to explore the biodiversity of each species among samples. Interestingly, strain distribution exposed a unique profile of prevalent PS between ICSTs, revealing patterns of multistrain coexistence specifically associated with either the Healthy-ICST or Unhealthy-ICST.

Reported ICST subdivision was further validated from an enzymatic perspective, highlighting extensive metabolic capabilities of the Unhealthy-ICST with respect to Healthy-ICST. The screening showed how opportunistic pathogens can rely on their enzymatic diversity to colonize the infant gut, while members of the indigenous microbiota possess a more compact and specialized enzymatic repertoire of genes. Nonetheless, the reported metabolic capabilities represent only the genomic and functional potential of these opportunistic pathogens since, through metatranscriptomic investigation, a different enzymatic expression pattern was revealed. Comparison between metagenomic and metatranscriptomic data highlights how a complex environment like the human gut must be investigated by employing both sequencing methodologies and possibly complemented with a metabolomics approach. While metagenomics analyses are very useful for microbial classification aimed at unveiling key players driving microbiota balances, the use of these data to understand the functional capabilities of the microbiota is not always warranted. Metatranscriptomic analyses allowed us to deduce enzymatic activities of microbial communities and provide reliable predictions of the metabolic activities of a microbial gut community, for example showing enhanced gene expression of genes related to amino acid biogenesis and vitamin precursors in Healthy-ICST.

## Methods

### Metagenome dataset selection

In this project, 10,935 publicly available datasets retrieved from 40 cohorts from various geographical locations were obtained through infant gut sequencing literature (Supplementary Table 1). To our best knowledge, at the time of writing of this manuscript, the collected metagenomic data represented the complete, publicly available biodiversity of the infant gut microbiota. In detail, we selected datasets of shotgun microbial profiling only, discarding all 16 S rRNA gene-related data, in an effort to achieve a detailed and reliable profiling of the microbiota at the species level. However, the selected datasets represented fecal samples belonging to infants with ages ranging from a few days following birth to 3 years. Therefore, no further exclusions were made based on the gathered metadata information, such as diet, type of birth, illness, probiotic, and antibiotic administration, in order to obtain a complete picture of the infant’s gut microbial biodiversity. Nonetheless, based on the collected metadata, three major groups were delineated, i.e., full-term healthy infants (HI), preterm newborns and critically ill infants (PI), and rural infants who do not belong to an urbanized country (RI).

### Taxonomic classification of the reads and whole-metagenome assembly

To analyze high-quality sequenced data only, each dataset was subjected to a filtering step removing low-quality reads (minimum mean quality score 20, window size 5, quality threshold 25, and minimum length 100) using the fastq-mcf script (https://github.com/ExpressionAnalysis/ea-utils/blob/wiki/FastqMcf.md). Filtered reads were then collected and taxonomically classified through the METAnnotatorX2 pipeline^29^, using the up-to-date RefSeq (genome) database retrieved from the NCBI (https://www.ncbi.nlm.nih.gov/refseq/). Filtered reads were then subjected to whole-metagenome assembly using Spades v3.15^30^ with default parameters and the metagenomic flag option (-meta) together with *k*-mer sizes of 21, 33, 55, and 77. As mentioned above, for the short reads, reconstructed contig sequences were taxonomically classified based on their sequence identity using megablast against the same RefSeq database^31^. ORFs of each assembled genome were then predicted with Prodigal^32^ and annotated utilizing the MEGAnnotator2 pipeline^33^. In all, the METAnnotatorX2 pipeline was employed for various purposes, from read filtering to taxonomic classification of the assembled contigs^29,34^.

### Infant community-state-type (ICST) prediction

Collected samples were subjected to hierarchical clustering (HCL) analysis based on their bacterial composition at the species level by means of Multiple Experiment Viewer (MeV) 4.8.1 software^35^. Average relative abundance data of seventeen species that displayed a prevalence between samples higher than 10% in conjunction with a prevalence higher than 3% as a major player (considering only the three most abundant species in each sample) were used to build clusters (Table 1). Person correlation was used as a distance metric based on the information of the microbial species abundances. Obtained data was represented by a cladogram that allowed the identification of eight ICSTs in the infant population screened in this project. The reference name attributed to each ICST was defined using the first two letters of the genus of those species with an average abundance higher than 10%, e.g., ICST-BI/EN is based upon *Bifidobacterium breve* and *Enterobacter hormaechei* (Fig. 2).

### Genome sequence selection of main ICST constituents

Complete and partial genomes of 44,987 bacterial strains were retrieved from the NCBI public database representing all sequenced genomes of the main ICST constituents. Furthermore, genome sequences of 16 reference strains was used to discard strains that do not belong to the actual species by using the 94% average nucleotide identity (ANI) threshold employing the software fastANI^36^. Using this approach, each bacterial strain employed in genomic analyses was verified avoiding misclassified microorganisms. Amino acid sequences of predicted proteins by the NCBI Prokaryotic Genome Annotation Pipeline (PGAP) system were then used for further genomic analyses^37^. Finally, the quality of reconstructed genomes from whole-metagenome assemblies was estimated for completeness and contamination using CheckM^38^.

### Metagenome tracing of main ICST constituents

Complete and partial genome sequences retrieved from NCBI together with those reconstructed from metagenomes were used to trace their presence among the 10,935 publicly available datasets collected in this study. First, to gather genomes with the highest average chromosomal coverage, 1700 samples in which the relative abundance of a single main ICST constituent was higher than 30% were subjected to WMS assembly using the METAnnotatorX2 pipeline^29^. Next, reconstructed genomes of main ICST constituents were selected based on statistics (completeness >50% and contamination <1.95%) retrieved using the CheckM software^38^. The contamination level cut-off of 1.95% was estimated by means of standard deviation (Whisker Range SD, Coef 1) using as input the contamination data collected from the 44,987 bacterial chromosome sequences retrieved from the NCBI public database, while the completeness cut-off of 50% was chosen arbitrarily to guarantee an adequate amount of genetic material to perform the analysis. Then, the distribution of each taxon was investigated using StrainGE software with a *k*-mer size of 23^39^. In detail, to select unique database representatives between 47,377 chromosomal sequences was used a *k-mer-based* clustering method at clustering genomes with an average nucleotide identity (ANI) higher than approximately 99.8%. Nonetheless, each reconstructed genome was previously validated using the 94% average nucleotide identity (ANI) threshold employing the software fastANI^36^, including the 2390 reconstructed genomes from the 10,935 publicly available datasets and the 44,987 downloaded genome sequences from NCBI.

### Functional profiling of main ICST constituents

Metagenomic datasets were screened against the MetaCyc metabolic database composed of 18,973 metabolites to retrieve each attributable enzymatic reaction^23^. The Enzyme Commission (EC) numbers were conferred to each nucleotide sequence by using Diamond^40^ in association with the METAnnotatorX2 pipeline^29^. Similarly, metatranscriptomic datasets of infant microbiomes were processed using an additional filtering step aiming in removing sequences belonging to rRNA and tRNA genes of the microbiota through BWA^41^ and a custom database including each sequence retrieved from the NCBI database^29^. The selection of 142 compounds was evaluated after detailed literature search efforts aimed at collecting health-related compounds that the gut microbiota can produce or metabolize. Subsequently, each screened EC has been associated with a predicted enzyme contained in a metabolic pathway (detailed in the MetaCyc Database), producing one or more compounds of interest.

### Statistical analysis

Bacterial abundance at the species level was validated by one-way ANOVA analysis. Post hoc analyses were performed using Tukey’s HSD (honestly significant difference) test. Furthermore, PERMANOVA analysis was performed using 1000 permutations to estimate *P* values of differences among infant samples in PCoA analyses. The hierarchical clustering analysis (HCA) of samples was performed using OriginPro graphing and analysis 2021 (https://www.originlab.com/2021), employing the Bray–Curtis matrix and Pearson correlation as a distance metric and the sum square of distances and furthest neighbor for clustering methods. The optimal number of clusters was defined through a Silhouette analysis. Moreover, multivariate analyses were performed through MaAsLin2 software^42^.

## Supplementary information

Supplementary Tables

Supplementary Figure 1

## Data Availability

Raw sequences of shotgun metagenomics datasets are accessible through SRA BioProjects listed in Supplementary Table 1, while metatranscriptomic datasets are reported in Supplementary Table 8. The source data underlying Figs. 1a, b, 2, 3a, b, 4, 5a–f, and Supplementary Fig. 1a, b are provided as a Source Data file.
