# Supplementary material for: Comprehensive insights from composition to functional microbe-based biodiversity of the infant human gut microbiota
Source: NPJ Biofilms Microbiomes. 2023 May 11;9:25. doi: 10.1038/s41522-023-00392-6 (PMC10175488; doi:10.1038/s41522-023-00392-6)
Supplement: Supplementary file 2 — Supplementary Figure 1 [file 41522_2023_392_MOESM2_ESM.pdf]

**a)**

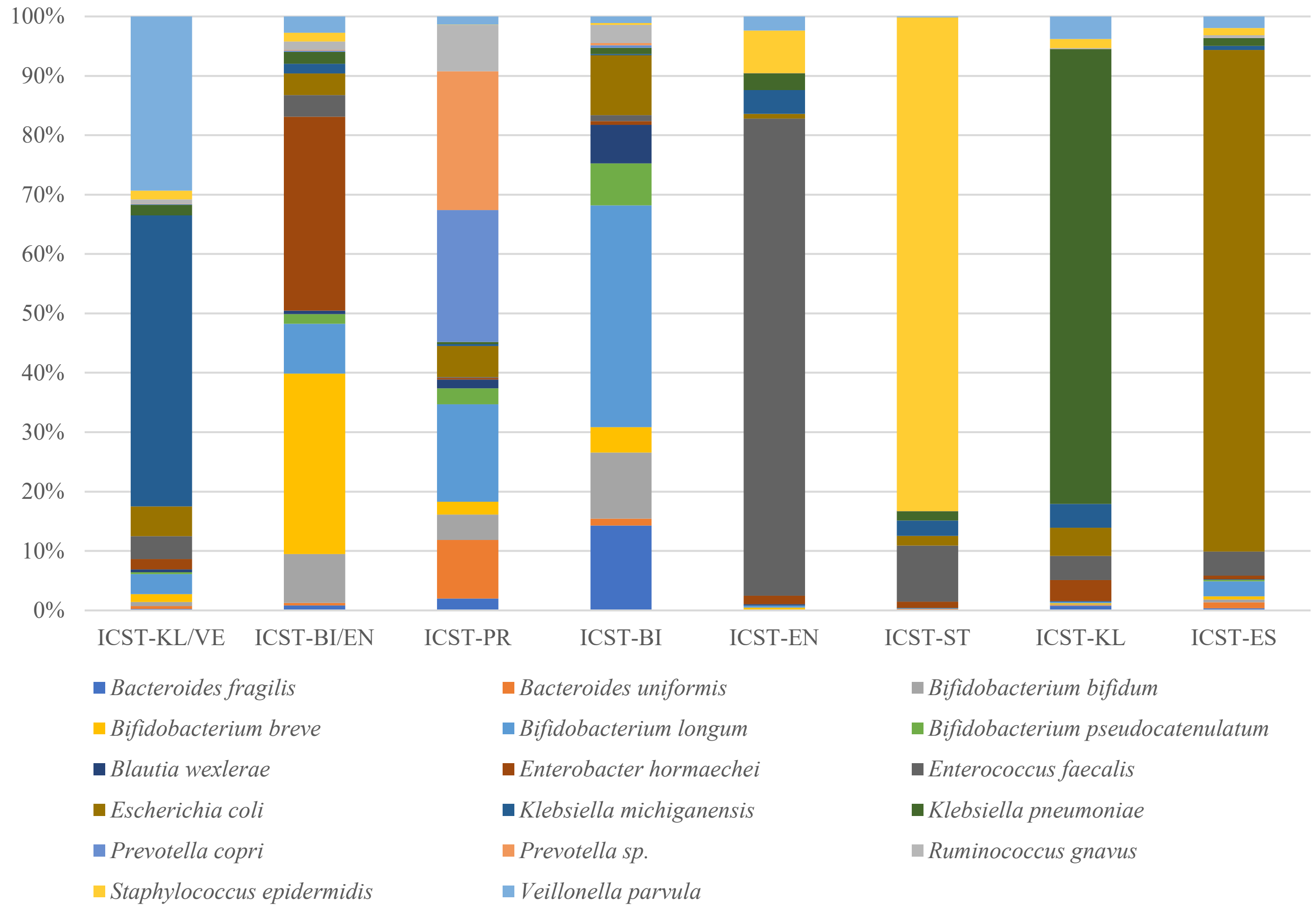


**b)**

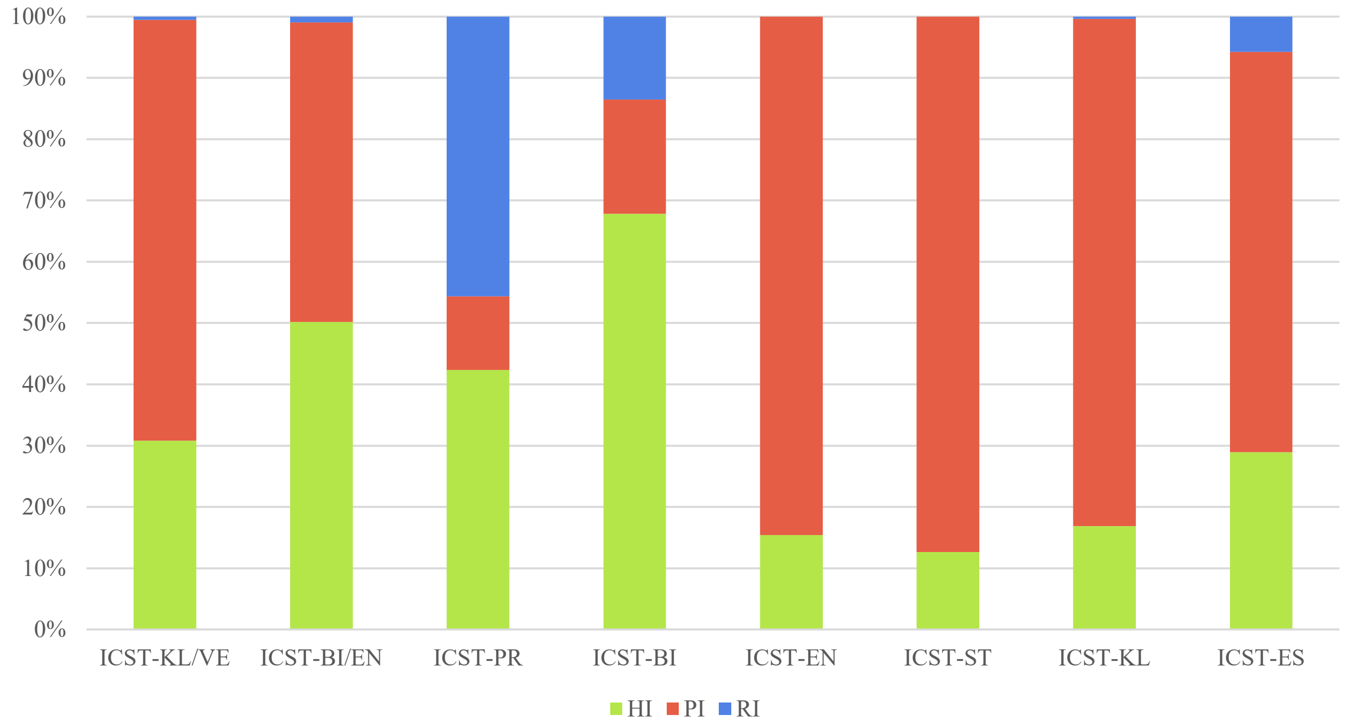


**Supplementary Figure 1.** Microbial composition of ICST groups. Panel a shows the relative abundances of main microbial species in each ICST cluster, while panel b exhibits the distribution of each ICTS between healthy, preterm, and rural infant clusters.
